# Increased extracellular volume in asymptomatic cocaine abusers detected by cardiovascular magnetic resonance imaging

**DOI:** 10.1186/1532-429X-15-S1-E101

**Published:** 2013-01-30

**Authors:** Ulf K Radunski, Ulrike Fuger, Jens Reimer, Gunnar Lund, Gerhard Adam, Stefan Blankenberg, Kai Muellerleile

**Affiliations:** 1 University Heart Center Hamburg, Hamburg, Germany; 2 Diagnostic and Interventional Radiology, University Medical Center Hamburg-Eppendorf, Hamburg, Germany; 3 Department of Psychiatry and Psychotherapy, University Medical Center Hamburg-Eppendorf, Hamburg, Germany

## Background

Cocaine abuse is associated with an increased risk for coronary artery disease and myocardial infarction. However, there is a paucity of data on myocardial injury in asymptomatic cocaine abusers. T1 mapping cardiovascular magnetic resonance (CMR) has the ability to quantify diffuse alterations in myocardial tissue composition by assessing the extracellular volume fraction (ECV). This study aimed at detecting silent myocardial injury in cocaine abusers using CMR.

## Methods

CMR was performed in eleven cocaine abusers and eleven matched controls without a history of cardiovascular disease. CMR protocol consisted of standard cine-, T2-STIR- and late gadolinium enhancement (LGE) CMR sequences to assess cardiac volumes and function, myocardial edema and focal myocardial fibrosis, respectively. Myocardial extracellular volume fraction (ECV) was assessed using a T1 mapping sequence (MOLLI) before and after administration of 0.075 mmol/kg gadolinium BOPTA: T1 maps were calculated with a dedicated plug-in written for the OsiriX software. Relaxation rates (1/T1 = R1) were calculated for myocardium and blood pool. The difference in R1 between pre- and post contrast media was calculated as ΔR1. Myocardial ECV was then estimated using the formula: ECV = 1-hematocrit * (ΔR1_myocardium_/ΔR1_blood pool_).

## Results

**Figure 1:**
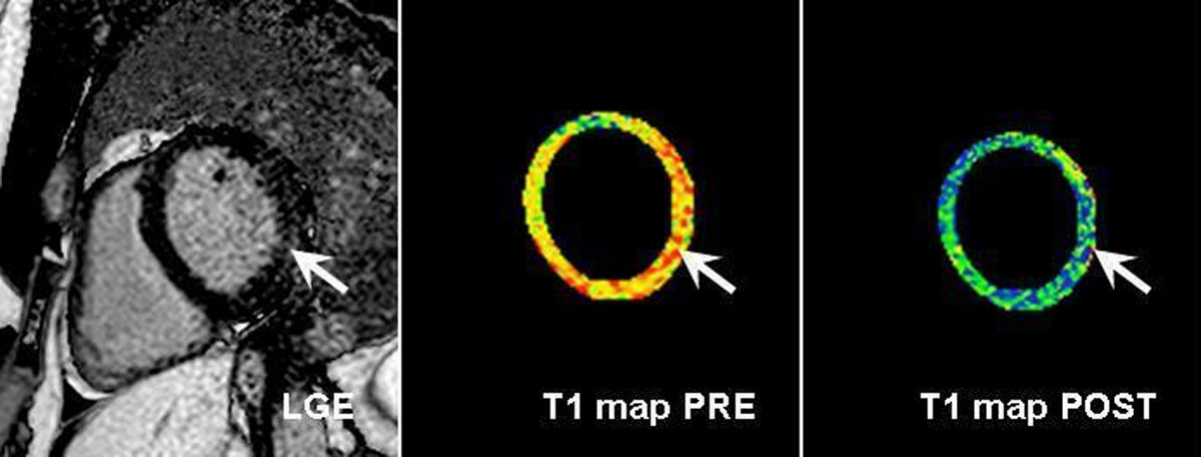


No significant difference was found in left ventricular ejection fraction between cocaine abusers and controls (58±7 % vs. 59±8 %; p=0.89). Neither cocaine abusers nor controls had myocardial edema on T2-STIR images. Discrete LGE with non-ischemic pattern was found in three cocaine abusers (27%) but not in controls (p=0.21). Global ECV was significantly larger in cocaine abusers compared to controls (31±6 % vs. 26±2 %; p<0.05). Figure 1 demonstrates an example with increased T1 values pre- vs. shortened T1 values post-contrast and the corresponding LGE image in a patient with infero-lateral LGE (arrow) and a global ECV of 32 %.

## Conclusions

Our findings indicate an increased myocardial extracellular space in asymptomatic cocaine abusers. These subtle myocardial alterations could represent early diffuse myocardial fibrosis and herald future cardiovascular events.

## Funding

None.

